# Aerosolization Behaviour of Fungi and Its Potential Health Effects during the Composting of Animal Manure

**DOI:** 10.3390/ijerph19095644

**Published:** 2022-05-06

**Authors:** Ruonan Wang, Aoyuan Yu, Tianlei Qiu, Yajie Guo, Haoze Gao, Xingbin Sun, Min Gao, Xuming Wang

**Affiliations:** 1 College of Forestry, Northeast Forestry University, Harbin 150040, China; loumeier@163.com (R.W.); yuaoyuan36@126.com (A.Y.); 2 Beijing Key Laboratory of Agricultural Genetic Resources and Biotechnology, Beijing Agro-Biotechnology Research Center, Beijing Academy of Agriculture and Forestry Sciences, Beijing 100097, China; qiutianlei@baafs.net.cn (T.Q.); guoyajie2007@163.com (Y.G.); ghz220414@163.com (H.G.); wangxuming@baafs.net.cn (X.W.)

**Keywords:** composting facility, airborne fungi, pathogenic/allergenic genera, aerosolization behaviour, factor analysis

## Abstract

Compost is an important source of airborne fungi that can adversely affect occupational health. However, the aerosol behavior of fungi and their underlying factors in composting facilities are poorly understood. We collected samples from compost piles and the surrounding air during the composting of animal manure and analyzed the aerosolization behavior of fungi and its potential health effects based on the fungal composition and abundance in two media using high-throughput sequencing and ddPCR. There were differences in fungal diversity and richness between the air and composting piles. Ascomycota and Basidiomycota were the two primary fungal phyla in both media. The dominant fungal genera in composting piles were Aspergillus, Thermomyces, and Alternaria, while the dominant airborne fungal genes were Alternaria, Cladosporium, and Sporobolomyces. Although the communities of total fungal genera and pathogenic/allergenic genera were different in the two media, fungal abundance in composting piles was significantly correlated with abundance in air. According to the analysis on fungal composition, a total of 69.10% of the fungal genera and 91.30% of pathogenic/allergenic genera might escape from composting pile into the air. A total of 77 (26.64%) of the fungal genera and six (20%) of pathogenic/allergenic genera were likely to aerosolize. The influence of physicochemical parameters and heavy metals on the aerosol behavior of fungal genera, including pathogenic/allergenic genera, varied among the fungal genera. These results increase our understanding of fungal escape during composting and highlight the importance of aerosolization behavior for predicting the airborne fungal composition and corresponding human health risks in compost facilities.

## 1. Introduction

The increase of centralized animal feeding operations (CAFOS) in many locations has increased the amount of manure produced [1]. Composting is an important method for recycling and stabilizing animal manure [2]. This biochemical process involves the interaction of diverse microbial communities to convert organic wastes into nutrient-rich, safe, and stable fertilizers and soil amendments [3,4]. Fungi play an important role in composting due to their ability to use many carbon substrates as a food source and attack organic residues that are too dry, acidic, or low in nitrogen for bacterial decomposition [5]. However, some operations involving vigorous movement during composting, such as moving and handling the compost material, are associated with the release of large amounts of bioaerosols [5,6,7,8]. Exposure to aerosolized microorganisms can have adverse effects on human health [9]. Fungal bioaerosols consist of spores, mycelium fragments, and debris which are easily inhaled by workers and cause numerous symptoms including allergies, irritation, and opportunistic infections. Long-term lung exposure to fungal bioaerosols can be associated with chronic diseases while the effects of short-term exposure range from irritation of the eyes and nose to coughing and a sore throat [10,11]. Given the inhalation of fungi can promote human health issues [10,11,12,13,14,15], there have been many studies on the abundance and composition of fungi in the air of composting sites [6,8,14,16].

However, there is little information on airborne fungi generated during the composting of animal manure [17,18] and this hampers the assessment of the environmental health risks under these conditions. The occupational health effects of airborne fungi may be underestimated since the worst fungal infections are usually caused by only a few species [19]. Traditional culture-based methods, fluorescence, and scanning electron microscopy cannot be used to effectively describe the entire fungal profile, and undetected fungal pathogens make it difficult to establish a definitive link between fungal exposure and respiratory problems [20]. High-throughput sequencing technologies have been used for the analysis of airborne fungi since they account for the relative abundance and full diversity of microorganisms present [17]. Accordingly, with the estimation of absolute abundance (EAA), we can obtain information on taxa abundance in the microbial communities [21,22,23]. The composition and quantification of fungi released from animal manure composting have not been previously studied, especially with regard to pathogens. This knowledge gap has prevented the quantitative assessment of human health risks from occupational exposure to airborne fungi.

Compost piles are important sources of airborne fungi [6]. Some fungi in piles are more prone to being aerosolized, and this preferential aerosolization could contribute to its widespread distribution in different air environments [24,25]. The aerosolization behavior of fungi was studied during the composting of vegetable waste [16] and during sewage sludge biostabilization [26] by comparing the fungal composition of air and its contributing sources. The aerosolization behavior of microorganisms in compost may be affected by their specific morphological, biochemical characteristics [24,27]. Other contributing conditions in compost piles include temperature, ventilation [28], as well as physico-chemical parameters [29], including heavy metals, exercising some influence on the microbial community [30]. However, the relationship between those potential factors and aerosolization of fungi, or fungal pathogens, is not known.

The objectives of this study were to investigate the aerosolization behavior of fungi and their potential factors during the composting of animal manure. The specific research goals included: (1) characterizing the concentration, diversity, and composition of fungi and pathogenic/allergenic genera and their potential health effects in air and composting piles; (2) describing the aerosol behavior of pathogenic/allergenic genera based on differences among key fungal genera, and connections of community between two media; (3) exploring the influence of physicochemical parameters and heavy metals on the aerosol index of fungal pathogenic/allergenic genera during composting. The present results will help us to understand the environmental risks and quantitatively assess human health issues posed by occupational exposure to airborne fungi during animal manure composting.

## 2. Materials and Methods

### 2.1. Sample Collection

We collected air and compost pile (C) samples from a commercial composting plant in Shouguang city, Shandong Province (118.50 N, 36.68 E) during thermophilic phases. Livestock manure, vegetables, and straws were the main raw materials used for compost production. In this study, totally 23 air samples and 12 solid samples were collected. Twelve and 11 air samples were collected from inside (I) (above the composting pile) and outside (O) the composting workshop, respectively, from 13 to 25 June 2020. Six solid samples were collected from the compost pile every two days during the same period.

We used total suspended particulate (TSP) air samplers (2030A, Laoying, Qingdao, China) to collect air onto a 90 mm (diameter) sterilized quartz-fiber filter (Ahlstrom Munktell, NO. 420065, Falun, Sweden) at a flow of 100 L/min for 24 h. Filters were baked in a muffle furnace at 500 °C for 5 h and stored in a sterilized plastic box until loaded into the sampler. Regarding each of the solid samples, 5 random samples were collected from the surface of compost pile using a sterile shovel before air collection. Those samples were mixed evenly and 100 g was taken for subsequent experiments. After sampling, both filters and composting piles were placed inside an ice chest, returned to the laboratory, and stored at −80 °C until analysis.

### 2.2. DdPCR and ITS Sequencing

For the TSP samples, 1/8 filter of each filter was used and cut into small pieces, which were loaded into Lysing Matrix E. A 0.3 g solid sample was loaded into Lysing Matrix E directly. DNA was extracted using the FastDNA^®^ SPIN Kit for soil (MP Biomedicals, Santa Ana, CA, USA) using manufacturer instructions. A Qubit^®^ dsDNA High Sensitivity Assay Kit (Life Technologies, Carlsbad, CA, USA) was used to measure the concentration of extracted DNA. All DNA samples were stored at −20 °C until analysis.

We used ddPCR to analyze the copy number of ITS in air and solids samples to determine the absolute abundance of fungi. ddPCR was run on a QX200 Droplet Digital™ PCR System (BioRad, Hercules, CA, USA). Each ddPCR reaction contained 20 μL of QX200 ddPCR EvaGreen Supermix (Bio-Rad), 100 nM of each primer, and 1 μL of sample DNA. The operating procedures and methods were the same as previously published [7], and the corresponding primers were ITS1F (5′-CTTGGTCATTTAGAGGAAGTAA-3′) and ITS2R (5′-GCTGCGTTCTTCATCGATGC-3′) [31]. PCR amplification of ITS was performed using the following conditions (heating rate 2.5 °C/s): 95 °C for 10 min, 40 cycles of 95 °C for 30 s, 55 °C annealing temperature for 60 s, and 72 °C 30 s at 4 °C, 5 min at 90 °C. After the PCR was completed, the 96-well plate was transferred to a Droplet Reader (Bio-Rad) for data collection. QuantaSoft™ software 1.7.4.0917 (Bio-Rad, Hercules, CA, USA) was used to automatically measure, record, and analyze the fluorescence per droplet and per well.

The internal transcribed spacer 1 (ITS1) region of the fungal rRNA gene was amplified using primers ITS1F and ITS2R through polymerase chain reactions (PCRs) [31]. The ITS sequencing was performed using Illumina MiSeq platform at Novogene Bio-Pharm Technology Co. Ltd. (Shanghai, China). Operational taxonomic units (OTUs) with a 97% similarity cutoff were clustered by UPARSE (Uparse v7.0.1001, http://drive5.com/uparse/, accessed on 24 May 2020) [32]. UCHIME was used to identify and remove chimeric sequences [33]. QIIME (version 1.9.0) [34] was used to assign taxonomy to OTUs based on the UNITE fungal ITS reference training data set for taxonomic assignment and to generate an OTU table.

### 2.3. Determination of Heavy Metals and Physical and Chemical Parameters

Concentrations of Cd were measured by a Graphite Furnace Atomic Absorption Spectrophotometer (GFAAS, HITACHI Z5000). Other heavy metals were determined using an Inductively Coupled Plasma-Atomic Emission Spectrometry (ICP-AES, PerkinElmer Optima 5300DV). Total nitrogen (N) was measured using an automatic N meter and Kjeldahl determination [35]. Total potassium (TK) and total phosphorus (TP) were measured according to NYT87-1988 and NYT88-1988 standard experimental methods, respectively. The organic matter (OM) in composting piles was determined as previously reported [36]. The pH was measured using a pH meter (pHS2F, Shanghai Precision and Scientific Instrument Co., Ltd., Shanghai, China). For air-dried moisture (AD), the solid samples were baked in a 105 °C incubator for about 2 h, moved to a desiccator to cool to room temperature. They were then weighed, and the AD value was calculated. A thermometer was used to determine the temperature of the compost pile every 48 h (Kedengbao Energy Technology TM-902C).

### 2.4. Data Analysis

The estimated absolute abundance (EAA) of certain fungal genera was calculated as the product of its relative abundance multiplied by the corresponding total copy number of ITS according to previous research on bacteria [21]. The relative abundance (*RA*) of a fungal taxon was determined by ITS sequencing and the copy number of airborne fungi was measured by ddPCR. The aerosolization behavior of microorganisms was quantified by the Bioaerosolization Index (*BI*) [24].

$$BI=\frac{RA_{aerosol}}{RA_{compost}},$$

where *RA* aerosol was the relative abundance (the percentage of a certain fungal genus among the total fungi) of certain fungi in the air and *RA* compost was the relative abundance of the corresponding fungus in the compost piles.

Box plots were drawn using the Origin Pro 8.5. One-way ANOVA was used to analyze the differences across the sampling settings by SPSS 26.0, and all statistical tests were considered significant at *p* < 0.05. A website (Draw Venn Diagram (ugent.be, accessed on 9 January 2022) was used to draw Venn diagrams. STAMP analysis was conducted using the Tukey–Kramer test to compare the statistical differences in fungal genera between different samples [37]. The correlation of fungal communities among the three media was analyzed using conditioned constrained principal coordinate analysis (CPCoA) and non-metric multidimensional scaling (NMDS), which were performed by the package “ape” and “ggplot2” based on the Bray–Curtis distance with R 4.1.2 (http://www.r-project.org/; accessed on 22 July 2021). The redundancy analysis diagram (RAD) was drawn using the webpage (http://www.cloud.biomicroclass.com/, accessed on 9 January 2022). Both Procrustes analysis (“vegan” and “labdsv” packages) and Heatmaps (“pheatmap” package) were drawn in the R environment.

## 3. Results and Discussion

### 3.1. Fungal Abundance and Diversity in Air and Compost Piles

**Figure 1:**
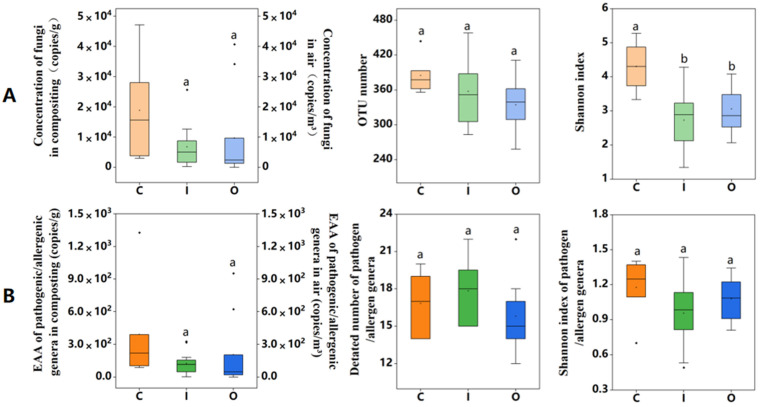
Abundance and diversity of fungal genera and pathogenic/allergenic genera in composting piles (C), inside air (I), and outside air (O) of composting workshop. (**A**) Concentration, OTU number, and Shannon indices of total fungal genera; (**B**) the estimation of absolute abundance (EAA), detected number, and Shannon indices of pathogenic/allergenic genera. (There is a significant difference between “a” and “b” above the box diagram; *p* < 0.05).

The fungal concentration (copies of ITS) and diversity (OTU number and Shannon index) in compost piles and air (inside and outside of the composting workshop) are presented in Figure 1A. The estimation of absolute abundance (EAA) was used for the quantitative analysis of 30 pathogenic/allergenic genera, and their biological indices are shown in Figure 1B.

The concentration of fungi in composting pile was 4.71 × 10^4^–2.99 × 10^3^ copies/g, and the corresponding concentration of airborne fungi from inside and outside composting workshop were 6.8 × 10^3^ and 9.721 × 10^3^ copies/m^3^, with concentration ranges of 2.72 × 10^2^–2.57 × 10^4^ and 24.88–3.41 × 10^4^ copies/m^3^, respectively (Figure 1A). The values of both richness and diversity of fungi in composting piles were higher than those in inside and outside composting workshop. Overall, the OTUs number (fungal richness) ranged from 258 to 458. Although the average value of OTUs observed in composting piles (385 ± 12.95) was higher than that in inside workshop (357 ± 58.04) and outside composting working shop (334 ± 42.84), there was no significant difference between the two air samples. The Shannon index of composting piles (4.31 ± 0.32) was significantly higher than that of inside (2.73 ± 0.26) and outside composting workshop (3.06 ± 0.21) (one-way ANOVA, *p* < 0.05), which is consistent with research on the composting of green waste (5 ± 0.1) [31].

The EAA value of airborne pathogenic/allergenic genera had a similar trend with the total fungi. A higher value was detected outside the composting workshop (2.05 × 10^3^ ± 0.92 × 10^3^ copies/m^3^) compared to the value inside (1.25 × 10^3^ ± 0.31 × 10^3^ copies/m^3^) (Figure 1B), but the difference was not statistically significant. The EAA of airborne pathogenic/allergenic genera was similar to biochemical and culture method test data from other composting facilities [18,38]. Our results confirmed the exposure risk of pathogenic/allergenic genera in the surrounding air environment during composting of animal manure. The number of pathogenic/allergenic genera from inside composting workshop (18) was slightly higher than that in composting piles (17) and outside composting workshop (16). No significant difference was detected on the Shannon index of pathogenic/allergenic genera among the three sampling settings in this study.

**Figure 2:**
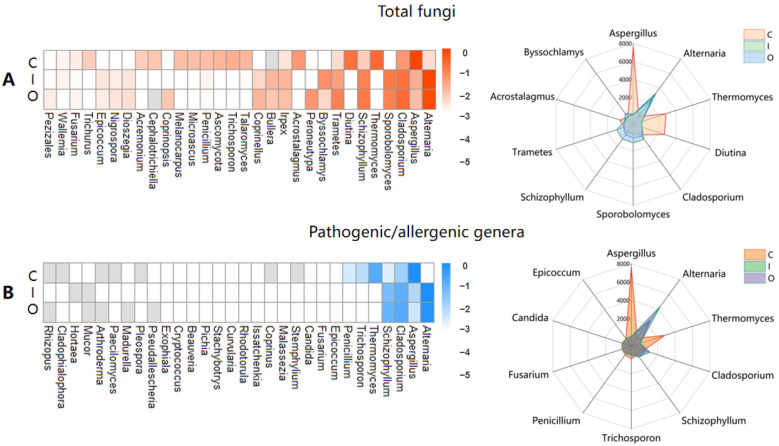
The dominant fungi and pathogenic/allergenic genera in compost piles (C), inside (I), and outside air (O) of composting workshop. (**A**), the relative abundance (Log value) of the top 30 dominant fungi by heat map and the estimation of absolute abundance (EAA) of 10 dominant fungi by radar map; (**B**), the relative abundance (Log value) of 30 pathogenic/allergenic genera by heat map and the EAA of 10 dominant pathogenic/allergenic genera by radar map.

We analyzed the dominant fungal phyla in the air (inside and outside air of composting workshop) and composting piles (Figure S1) and found that Ascomycota and Basidiomycota were consistently the two primary phyla in all three sample settings during thermophilic phases (Figure S2). The same dominant fungal phyla were detected in the composting of pig carcasses [6] and green waste [8], as well as sewage sludge biostabilization [26]. Given that Ascomycetes are major contributors to the degradation of holocellulose [39], they are commonly found in other composting processes as well as in this study due to the presence of straw in the raw materials. The relative abundance of Ascomycota in compost piles (86.96 ± 4.56%) was higher than their abundance in the air (61.77 ± 26.85%). However, a higher relative abundance of Basidiomycota was detected in the air (23.43 ± 22.29%) compared with abundance in compost piles (3.83 ± 2.75%). We analyzed the dominant fungal genera in 29 samples from compost piles, inside and outside the composting workshop (Figure S3). The average values of dominant fungal genera by relative abundance (heat map) and concentration (radar map) are shown in Figure 2A. The dominant fungi in compost piles were *Aspergillus* (45.28 ± 16.64%), *Thermomyces* (14.98 ± 11.27%), *Alternaria* (13.98 ± 11.27%), *Diutina* (10.30 ± 21.70%), and *Cladosporium* (1.26 ± 0.56%), while the dominant airborne fungal genes were from *Alternaria* (47.41 ± 9.62%), *Cladosporium* (11.57 ± 2.67%), *Sporobolomyces* (7.47 ± 1.37%), *Schizophyllum* (6.78 ± 1.44%), and *Aspergillus* (1.25 ± 0.42%). Distinctive distributions of airborne fungal compositions compared to those in compost have been previously reported [6,26]. The differences may be due to the different aerosolization behavior of fungi and different environmental pressures in the two media [40]. This difference may also be caused by differences in the biological mechanisms of spore ontogeny and spore release [41,42].

Advances in high-throughput sequencing and bioinformatics enable the study of fungal pathogenic/allergenic genera. *Alternaria*, *Aspergillus*, *Cladosporium*, *Schizophyllum*, and *Thermomyces* were the top five fungi with the highest relative abundance among the target 30 fungal pathogenic/allergenic genera (Figure 2B, heat map). Combined with the concentration of ITs, the quantitative information (expressed as EAA) of the top five pathogenic/allergenic genera were also analyzed (Figure 2B, radar map). According to the EAA, the highest concentration of airborne pathogenic/allergenic genera was detected from *Alternaria* (3.43 × 10^3^ ± 5.80 × 10^3^ copies/m^3^). The EAA of *Cladosporium* in the air was (8.82 × 10^2^ ± 1.08 × 10^3^ copies/m^3^), which was similar to previous studies [18,38,43,44]. *Aspergillus fumigatus* was the only specific taxon for which UK legislation requires compost site testing [45]. *A. fumigatus* has also been detected in the air of other composting facilities [8,27,31]. Because of infections caused by *A. fumigatus* [46], the considerable relative abundance (1.26 ± 0.86%) and EAA value (0.86 × 10^2^ ± 0.98 × 10^2^ copies/m^3^) of *Aspergillus* in the compost facility air is a health concern. The quantitative and qualitative data on pathogenic/allergenic genera in this study reveal possible human health risks from the air environment during the composting of animal manure.

The physicochemical parameters in the composting piles over 12 days are shown in Table 1. During this thermophilic stage, the temperature ranged from 68.5 and 72.5 °C, with an average value of 70.5 ± 1.35. The content of total nitrogen (TN) increased from 1.58 ± 0.00% to 2.02 ± 0.01%. Usually, the variation of TN was caused by the degradation of organic compounds and the metabolic processes of microorganisms. The similar trend was also detected in previous research [26]. Meanwhile, the increasing biological metabolism during the composting process [47] might also have resulted in the increase of organic matter as we found that OM% increased from 19.65 ± 0.12% to 30.05 ± 0.04%. Both AD and pH were relatively stable in the thermophilic stage, with mean values of 1.9 ± 0.10 and 8.72 ± 0.13, respectively. Seven heavy metals in the compost piles were also analyzed (Table 2). The highest heavy metal concentration was Zn (255.61 ± 1.56 mg/kg) and the lowest was Hg (0.04 ± 0.00 mg/kg). The concentration of Cu is within the range of the published literature [30].

### 3.2. Connection and Difference of the Fungal Community and Abundance between Composting Piles and Air

**Figure 3:**
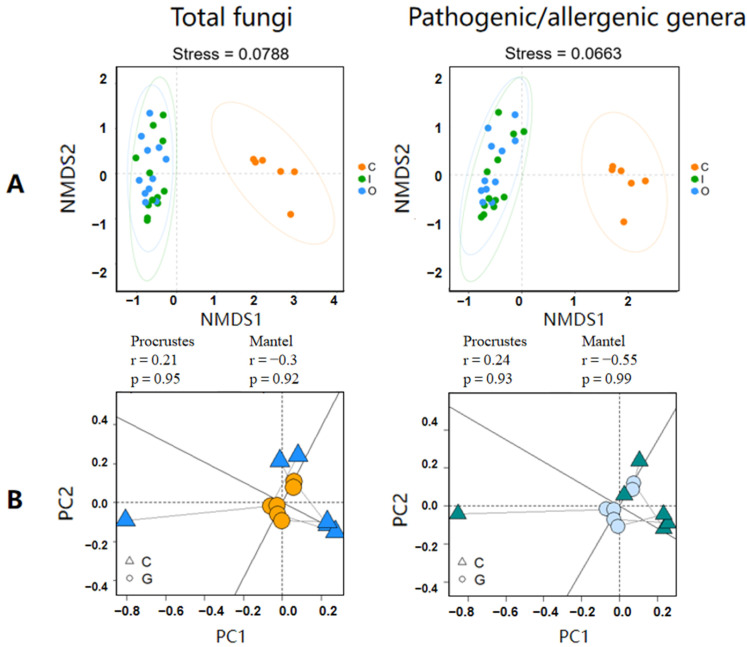
Relation of fungal community between composting piles and air by total fungal genera and pathogenic/allergenic genera. (**A**), fungal lineages used non-metric multidimensional scaling analysis (the orange point represents compost piles, the green represents the inside, and the blue represents outside air of composting workshop); (**B**), fungal community correlation used Procrustes and Mantel analysis (the triangle represents compost piles, and the circle represents the air sample).

We examined microbial community correlations between air and composting piles by total fungal genera and pathogenic/allergenic genera (Figure 3A,B).

Fungal populations in different sampling settings were clustered using non-metric multidimensional scaling (NMDS) analysis at the genus level based on the Bray–Curtis dissimilarity coefficient. Both the community of total fungal genera and pathogenic/allergenic genera in the compost piles were distinguished from those in the two air samples (Figure 3A). CPCoA also demonstrated the significance of this difference (*p* = 1 × 10^−4^ in Figure S4). The airborne fungal communities of air sampling settings were clustered closer compared with those in the compost piles. A similar distribution of clusters was also observed in pathogenic/allergenic fungal communities from the three sampling settings. This dissimilarity in fungal communities between air and compost was confirmed using Procrustes and Mantel analyses (Figure 3B).

Our study data suggest that the aerosol process of fungal communities is not a synchronized behavior of the overall fungi, and the aerosolization behavior of specific fungal genera may vary. In addition, the environmental stresses to which fungi are exposed in air and compost piles might also contribute to their different abundances and compositions [40]. Since the composition in compost piles was not sufficiently accurate to predict the escaped airborne fungal community and subsequent potential health risk, it is crucial to develop a targeted assessment of the aerosolization behaviors of specific fungi.

**Figure 4:**
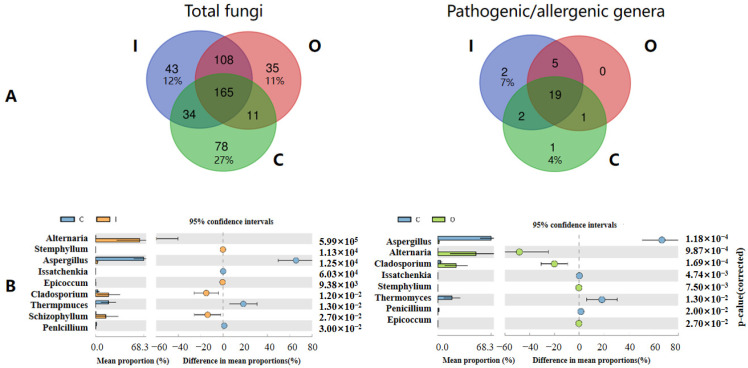
Common and endemic fungi in air and compost piles. (**A**) Number of common and endemic total fungi and pathogenic/allergenic genera in composting piles (C), inside (I), and outside air (O) of composting workshop using a Venn diagram, (**B**) Identification of different pathogenic/allergenic genera between C and I, or C and O using Stamp analysis. The bar chart shows the average proportion of differential pathogenic/allergenic genera predicted using Picrust 2. The difference in proportions between any two groups is presented with 95% confidence intervals. Only *p* < 0.05 (Welch’s *t* test, FDR adjusted) are depicted.

To explain the above distinguishing community, we analyzed specific fungal genera between air and compost piles, including the number of common and endemic fungal genera (Venn diagram in Figure 4A), and specific differences among fungal genera (Stamp analysis in Figure 4B).

A total of 78 endemic genera were detected in composting piles, followed by inside (43), and outside of composting workshop (35), with corresponding proportions (ratio of endemic genera to the total fungal genera) of 27%, 12%, and 11%, respectively. A total of 165 airborne fungal genera were shared across the three sampling settings. Compost piles shared more common fungal genera with inside (199) than outside workshops (176), and the most common fungal genera were detected in the two air sampling areas (273). Based on the fungal composition, 69.10% of fungal genera in compost piles could become airborne. Among the pathogenic/allergenic genera, only one endemic genus was detected in composting piles (*Mucor*). Consistent with fungal genera, the number of shared pathogenic/allergenic genera between composting piles and I was greater than between composting piles and O. Most (91.30%) of the pathogenic/allergenic genera in composting piles were shared with inside air, suggesting that most could aerosolize and emit into the air.

The key fungal genera driving the significantly different proportions between composting piles and I (Figure S5), and composting piles and O (Figure S6) were analyzed using Stamp analysis. More different genera were detected between composting piles and inside air (38) than between composting piles and outside air from composting workshop (33). Stamp analysis on the key pathogenic/allergenic genera (Figure 4B) showed that the key fungal genera from composting piles to inside air was slightly higher than that to outside air. Most of the critical genera were identical, except for *Schizophyllum* with significantly high relative abundance in inside air of workshop.

**Figure 5:**
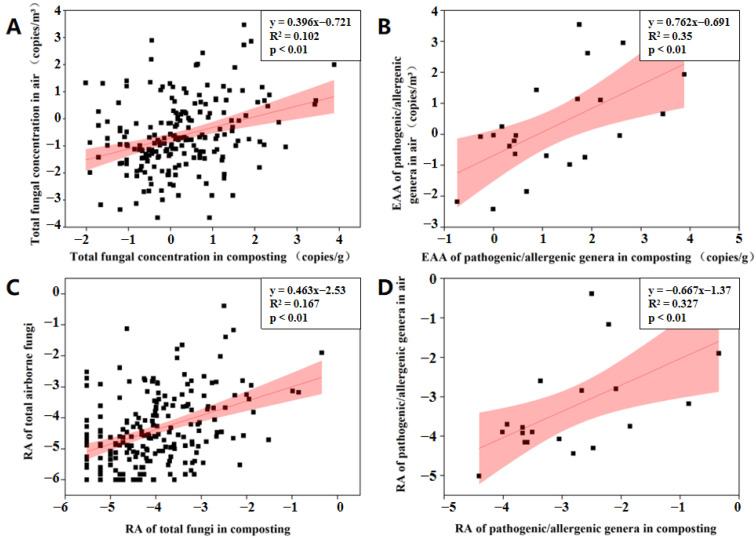
Correlation of fungal concentrations and relative abundances between air and compost piles using linear regression analysis. (**A**), total fungal concentrations; (**B**), the estimation of absolute abundance (EAA) of pathogenic/allergenic genera; (**C**), Relative abundance of total fungi; (**D**), Relative abundance of pathogenic/allergenic genera. Data were log-transformed.

To quantitatively assess fungal aerosolization behavior, we analyzed the correlation of fungal concentrations and relative abundances in compost piles and air using linear regression analysis. The total fungal concentration in air was significantly correlated with that in composting workshop (R^2^ = 0.102, *p* < 0.01) (Figure 5). We also detected significant correlations between pathogenic/allergenic genera in air and compost media based on EAA values (R^2^ = 0.35, *p* < 0.01). The relative abundance of both total fungi and pathogenic/allergenic genera in the air also increased with the increase of the corresponding value in compost piles (*p* < 0.01). Most research on fungal aerosolization behavior has focused on the relative abundance of specific fungal genera between air and compost [6,26] Based on concentrations and relative abundances, we established a link between the biomass of airborne fungi (including the pathogenic/allergenic genera) and its potential source (compost). This can help predict the quantitative trends of airborne fungi according to their levels in compost piles.

### 3.3. Potential Factors Affecting the Aerosolization Behavior of Airborne Fungi and Pathogenic/Allergenic Genera

The aerosolization behavior of each specific fungal phylum or genus was described using the Bioaerosolization Index (BI). A log BI value greater than 0 indicates that the fungus is more likely to spread from the compost into the air and vice versa. The aerosolization behavior of fungal phyla was analyzed and the results are shown in Figure S7. Although the Ascomycota and Basidiomycota were the most dominant fungal phyla in both air and compost piles, they have different aerosolization behaviors. Basidiomycota (log BI = 0.798 ± 0.42) had preferential aerosolization while Ascomycota (log BI = −0.16 ± 0.22) represented passively aerosolized microorganisms. A similar trend in the aerosolization behavior of the same fungal phyla was observed during the composting of vegetable waste [16] and sewage sludge biostabilization [26]. 

**Figure 6:**
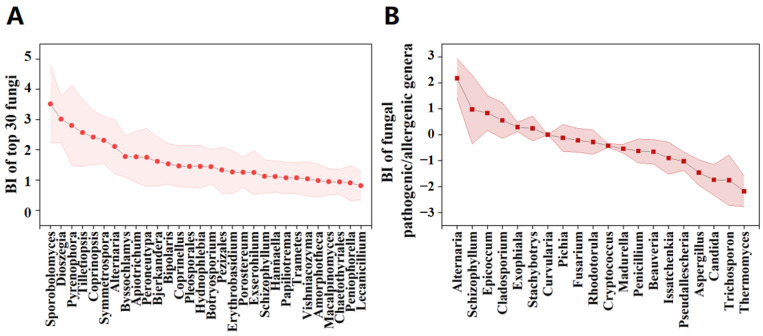
Bioaerosolization Index (BI) of the 30 top fungal genera (**A**) and pathogenic/allergenic genera (**B**) (BI value was t log-transformed).

The results on aerosolization behavior of fungal genera (Figure S8) indicated that 26.64% of all fungi were easily spread from composting piles into the surrounding air with log BI value being higher than zero. The highest value of log BI (*Sporobolomyces* of 3.94) was comparable to previous studies [16,26]. The number of preferential fungus genera (77) was higher than that in the composting of vegetable waste (7) [16] and sewage sludge biostabilization (11) [26], suggesting that a greater number of fungi genera in manure compost are likely to aerosolize. The five most easily aerosolized fungi were *Sporobolomyces* (BI = 3.52 ± 1.24), *Dioszegia* (BI = 2.97 ± 0.89), *Pyrenophora* (BI = 2.77 ± 0.51), *Tilletiopsis* (BI = 2.53 ± 0.24), and *Coprinopsis* (BI = 2.53 ± 0.24). Similar to the results of fungal phyla, the five dominant fungal genera (Figure 2) were not completely consistent with the top five genera with preferential aerosolization (Figure 6) (except *Sporobolomyces*). This discrepancy here, as well as in previous studies [16,26], indicates that detailed studies are needed to determine the underlying mechanisms of fungal aerosolization behavior.

The aerosolization behavior of pathogenic/allergenic genera is shown in Figure 6B. Among the 30 target pathogenic/allergenic genera, the log BI values of six genera (20%) were greater than 0. There was species-specific aerosolization behavior in the pathogenic/allergenic genera (Figure 6B). Although the enrichment of *Aspergillus* in the air was reported in both animal and domestic composting facilities [6,16], its log BI (log BI = −1.56 ± −1.53) value here was lower than 0. This result indicates that, in addition to the characteristics of the fungus itself, other factors might play a non-ignored role on fungal aerosolization behavior. Extensive exposure to mycotoxins produced by *Fusarium* may cause respiratory symptoms in humans [48]. *Fusarium* (log BI around 3) was the most preferred fungal genus for aerosolization during biostabilization [26]. In this study, we found it to be a passively aerosolized microorganism with log BI = −0.17 ± 0.093. Our results suggest that the aerosolization behavior of some fungal genera might differ between different composting facilities.

**Figure 7:**
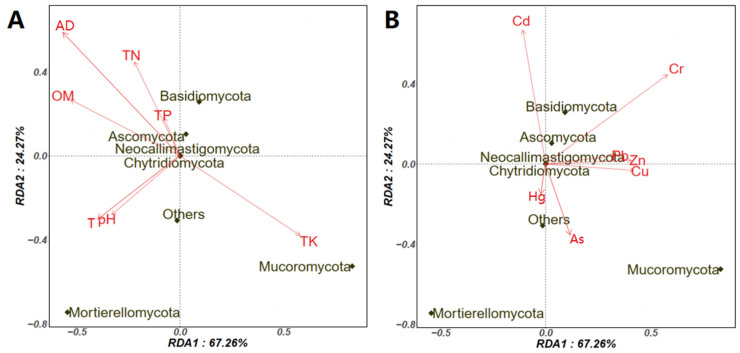
Factors involved in the aerosolization behavior of fungal phylum using redundancy analysis (RDA). (**A**), physicochemical parameters on log BI of fungal phylum; (**B**), Specific heavy metals on log BI of the fungal phylum. OM, Organic matter; AD, air-dried moisture; TN, Total nitrogen; TK, Total potassium; TP, Total phosphorus; OM, The organic matter; AD, Air-dried moisture; T, Temperature.

To explore the potential factors related to the aerosolization behavior of fungi, we analyzed the relation of physicochemical parameters (Table 1) and specific heavy metals (Table 2) with the log BI of preferentially aerosolized fungi using redundancy analysis (RDA). According to the RDA result on the fungal phylum (Figure 7), AD, TN, TP, and OM had a positive effect on the aerosolization behavior of most fungal phyla, including *Ascomycota*, *Basidiomycota*, *Chytridiomycota*, and *Neocallimastigomycota*. The heavy metals Cd, Cr, Pb, Zn, and Cu promote the aerosolization behavior of *Ascomycota*, *Basidiomycota*, *Chytridiomycota*, and *Neocallimastigomycota*. The effect of both physicochemical parameters and heavy metals on the above four fungal phyla were different from the effects on *Mucoromycota* and *Mortierellomycota*. Although the heavy metals, such as Cu and As, have been reported to limit the growth and diversity of the microbial communities during manure composting [30], we found that Cu promotes the aerosolization behavior of certain fungal phylum. Current results highlight that future study should be conducted regarding the detailed correlation between heavy metal and specific fungal phylum in composting piles, as well as their influence on the aerosolization behavior.

**Figure 8:**
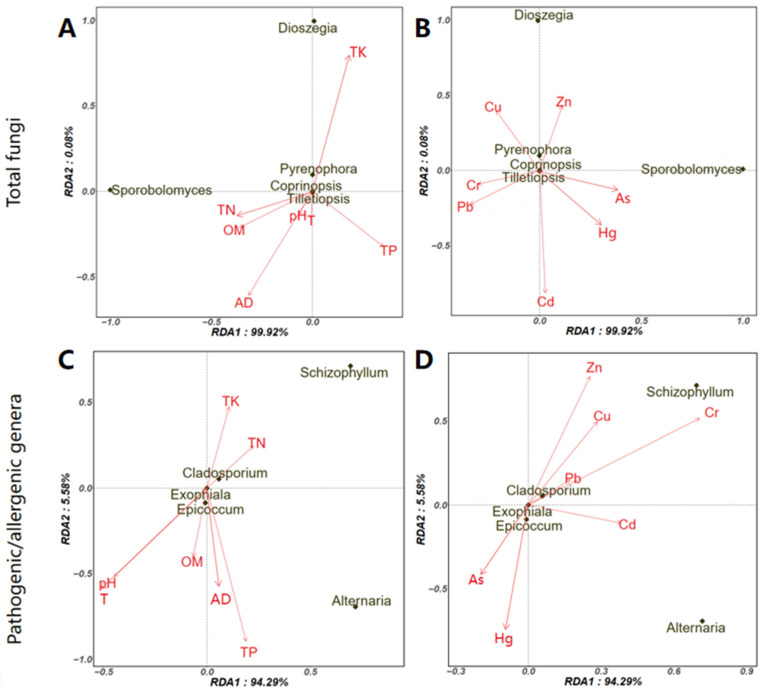
Factors associated with the aerosolization behavior of fungal genera using redundancy analysis. (**A**), physicochemical parameters on top 5 fungi with the highest log BI; (**B**), Specific heavy metal on top 5 fungi with the highest log BI; (**C**), physicochemical parameters on top 5 pathogenic/allergenic genera with the highest log BI; (**D**), Specific heavy metal on top 5 pathogenic/allergenic genera with highest log BI.

Figure 8 shows the potential influence of physicochemical parameters and heavy metals on the aerosolization behavior of the five fungal genera with the highest log BI value. These included *Sporobolomyces*, *Dioszegia*, *Pyrenophora*, *Tilletiopsis,* and *Coprinopsis* (Figure 6). Most previous studies focused on the effects of physicochemical factors on microorganisms in compost piles [49,50]. To our best knowledge, no studies determined their influences on aerosolization behavior. We found that the TK or TN was positively correlated with at least three fungi (Figure 8A), and a similar trend was found in pathogenic/allergenic genera (Figure 8C). TN is an important nitrogen source that supports microbial activity and increases the growth of fungal communities in piles [50], which might have a potential effect on their aerosolization behavior. Although the adjustment of moisture content (AD) might eliminate the bioaerosol emission of hydrophilic microorganisms (e.g., fungi) [26], both the positive and negative effect of AD on fungal aerosolization behavior were detected here and depended on the specific fungal genus. The concentration of airborne bacteria emissions in the composting of swine manure was positively correlated with the temperature in the compost pile [51]. We also found that temperature facilitated aerosolization of *Tilletiopsis*, *Coprinopsis*, and *Epicoccum*. The heavy metals Zn and Cu seem to promote the aerosolization process of *Dioszegia*, *Pyrenophora*, *Tilletiopsis,* and *Coprinops* (Figure 8B) as well as the pathogenic/allergenic genera, including *Schizophyllum*, *Cladosporium*, and *Exophiala* (Figure 8D).

We found no consistent influence of a specific factor on fungal aerosolization including physicochemical parameters or heavy metals. This may be because factors other than those tested here may be involved in fungal aerosolization. Understanding the mechanisms involved during aerosol emission will require studies on a broader range of potential factors, including compost age, activity, or material composition [25]. The capability of fungi to become aerosolized may also depend on their morphological characteristics (form, structure [52], and size [16]), and on their biochemical characteristics, such as hydrophobicity, which vary significantly among species [24,25,27]. These factors also need to be evaluated in future research.

## 4. Conclusions

Fungi in compost can be aerosolized, resulting in adverse effects on occupational health. In this study, we analyzed the aerosol behavior of fungi and their underlying factors in composting facilities. Our results indicate that, due to differences in the community structure in air and compost, the fungal composition in compost piles could not accurately predict the composition of airborne fungi. However, most fungal genera and pathogen/allergen containing genera in compost might potentially aerosolize into the air, and about 25% of these are likely to aerosolize. The influence of physicochemical parameters and heavy metals on the aerosol behavior of fungi, including pathogenic/allergenic genera, varied depending on the fungal genus evaluated. Current results on the compositions and abundance of pathogenic/allergenic genera highlight the possible risks to human health posed by the air environment during animal manure composting. The link between the biomass of airborne fungi (including the pathogenic/allergenic genera) and its potential source (compost) can help predict the quantitative trends of airborne fungi and corresponding health risk according to their levels in compost piles.

## Figures and Tables

**Table 1:** Physicochemical parameters in composting piles.

*Time*	*TK (%)*	*TN (%)*	*TP (%)*	*OM (%)*	*pH*	*AD (%)*	*T* (°C)
*2020.06.14*	6.49 ± 0.01	1.58 ± 0.00	0.73 ± 0.00	19.65 ± 0.12	8.93 ± 0.03	1.90 ± 0.00	72.50 ± 0.00
*2020.06.16*	6.31 ± 0.00	1.80 ± 0.01	0.70 ± 0.00	33.70 ± 0.16	8.67 ± 0.03	2.00 ± 0.00	72.00 ± 0.00
*2020.06.18*	6.16 ± 0.02	1.81 ± 0.01	0.76 ± 0.00	28.85 ± 0.04	8.69 ± 0.00	1.90 ± 0.00	70.00 ± 0.00
*2020.06.20*	5.98 ± 0.00	1.90 ± 0.00	0.80 ± 0.00	27.30 ± 0.08	8.50 ± 0.02	1.70 ± 0.00	68.50 ± 0.00
*2020.06.22*	6.48 ± 0.03	2.01 ± 0.01	0.81 ± 0.00	28.10 ± 0.08	8.78 ± 0.04	1.90 ± 0.00	70.00 ± 0.00
*2020.06.24*	6.01 ± 0.02	2.02 ± 0.01	0.67 ± 0.00	30.05 ± 0.04	8.74 ± 0.03	2.00 ± 0.00	70.00 ± 0.00
*Mean value*	6.24 ± 0.21	1.85 ± 0.15	0.75 ± 0.05	27.94 ± 4.24	8.72 ± 0.13	1.90 ± 0.00	70.50 ± 0.00

Organic matter (OM); air-dried moisture (AD); Total nitrogen (TN); Total potassium (TK); Total phosphorus (TP); The organic matter (OM); Air-dried moisture (AD); Temperature (T). “±” stands for “SD”.

**Table 2:** Concentration of heavy metals in composting piles (mg/kg).

*Time*	*Pb*	*Cd*	*Cr*	*Cu*	*Zn*	*Hg*	*As*
*2020.06.14*	18.05 ± 0.58	1.37 ± 0.02	20.42 ± 0.03	41.80 ± 0.08	231.26 ± 4.68	0.06 ± 0.00	2.03 ± 0.07
*2020.06.16*	14.09 ± 0.87	1.12 ± 0.04	16.66 ± 0.25	44.32 ± 0.24	223.14 ± 0.91	0.06 ± 0.00	1.80 ± 0.01
*2020.06.18*	14.81 ± 0.81	1.13 ± 0.02	20.60 ± 0.15	49.62 ± 0.04	277.25 ± 0.35	0.03 ± 0.00	1.72 ± 0.06
*2020.06.20*	15.93 ± 0.12	1.33 ± 0.01	31.56 ± 0.09	49.55 ± 0.18	278.00 ± 2.48	0.03 ± 0.00	1.33 ± 0.01
*2020.06.22*	14.55 ± 0.58	1.26 ± 0.01	22.83 ± 0.13	48.27 ± 0.01	253.77 ± 0.92	0.03 ± 0.00	1.30 ± 0.02
*2020.06.24*	13.81 ± 0.99	1.26 ± 0.05	19.36 ± 0.19	46.39 ± 0.19	270.23 ± 0.01	0.01 ± 0.00	1.20 ± 0.01
*Mean value*	15.20 ± 0.66	1.25 ± 0.02	21.90 ± 0.14	46.66 ± 0.12	255.61 ± 1.56	0.04 ± 0.00	1.56 ± 0.03

## Data Availability

Not applicable.

## Supplementary Materials

The following supporting information can be downloaded at: https://www.mdpi.com/article/10.3390/ijerph19095644/s1, Figure S1: Top 10 dominant fungal phyla in compost piles (C), inside (I) and outside (O) composting workshop; Figure S2: Heatmap of relative abundance of the top 10 dominant fungal phyla in compost piles (C), inside (I) and outside (O) composting workshop; Figure S3: Heatmap of relative abundance of the top 30 dominant genera of fungi in composting piles (C), inside (I) and outside (O) composting workshop; Figure S4: Cluster analysis results of fungal community in samples from compost piles (C), inside (I) and outside (O) composting workshop; Figure S5: Identification of different fungal genera between compost piles (C) and inside composting workshop (I) using Stamp analysis. The bar chart depicts the average proportion of differential pathogen/allergen containing genera predicted using Picrust2. The difference in proportions between any two groups is presented with 95% confidence intervals. Only *p* values < 0.05 (Welch’s *t*-test, FDR adjusted) are depicted; Figure S6: Identification of different fungal genera between compost piles (C) and outside composting workshop (O) using Stamp analysis. The bar chart depicts the average proportion of differential pathogen/allergen containing genera predicted using Picrust2. The difference in proportions between any two groups is presented with 95% confidence intervals. Only *p* values < 0.05 (Welch’s *t*-test, FDR adjusted) are depicted; Figure S7: Bioaerosolization Index (BI) of fungal phylum. (BI value was transformed by log); Figure S8: Bioaerosolization Index (BI) of 77 fungal genera with a BI value greater than 0. (BI value was transformed by log).
