# Supplementary material for: Aerosolization Behaviour of Fungi and Its Potential Health Effects during the Composting of Animal Manure
Source: Int J Environ Res Public Health. 2022 May 6;19(9):5644. doi: 10.3390/ijerph19095644 (PMC9101844; doi:10.3390/ijerph19095644)
Supplement: Supplementary file 1 [file ijerph-19-05644-s001.zip › ijerph-1652710-supplementary.pdf]

Supporting Information

Aerosolization Behavior of Fungi and Its Potential Health Risks during the Composting of Animal Manure

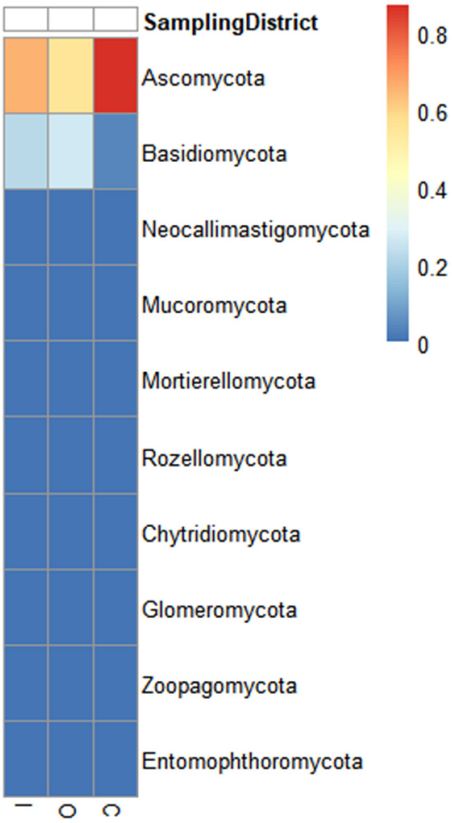

Figure S1 Top 10 dominant fungal phyla in compost piles (C), inside (I) and outside (O) composting workshop

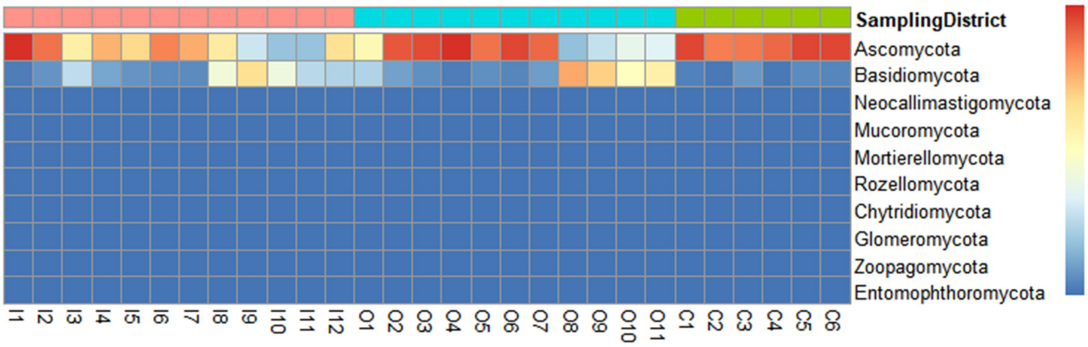

Figure S2 Heatmap of relative abundance of the top 10 dominant fungal phyla in compost piles (C),

inside (I) and outside (O) composting workshop

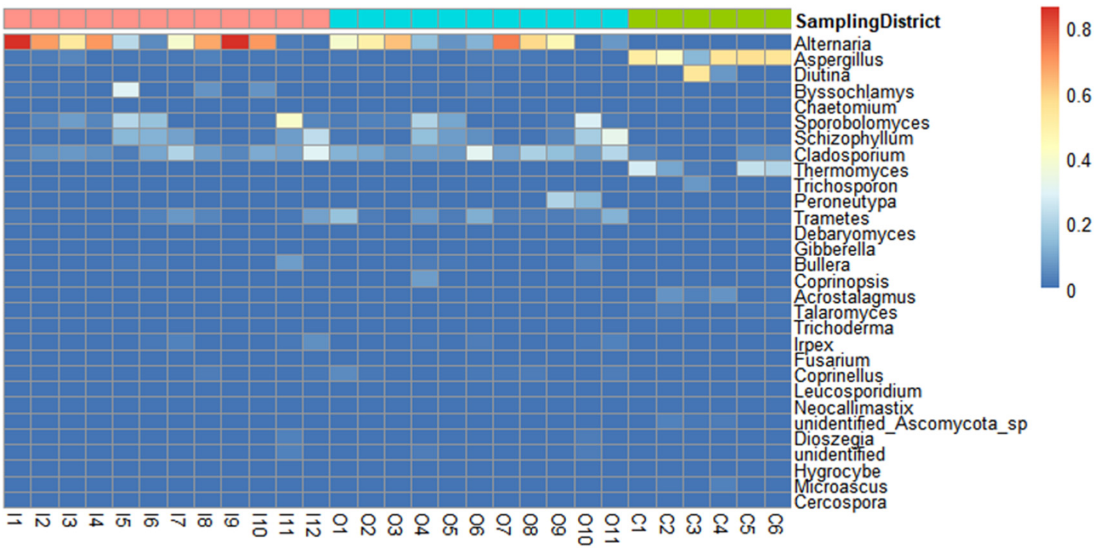

Figure S3 Heatmap of relative abundance of the top 30 dominant genera of fungi in composting piles (C), inside (I) and outside (O) composting workshop

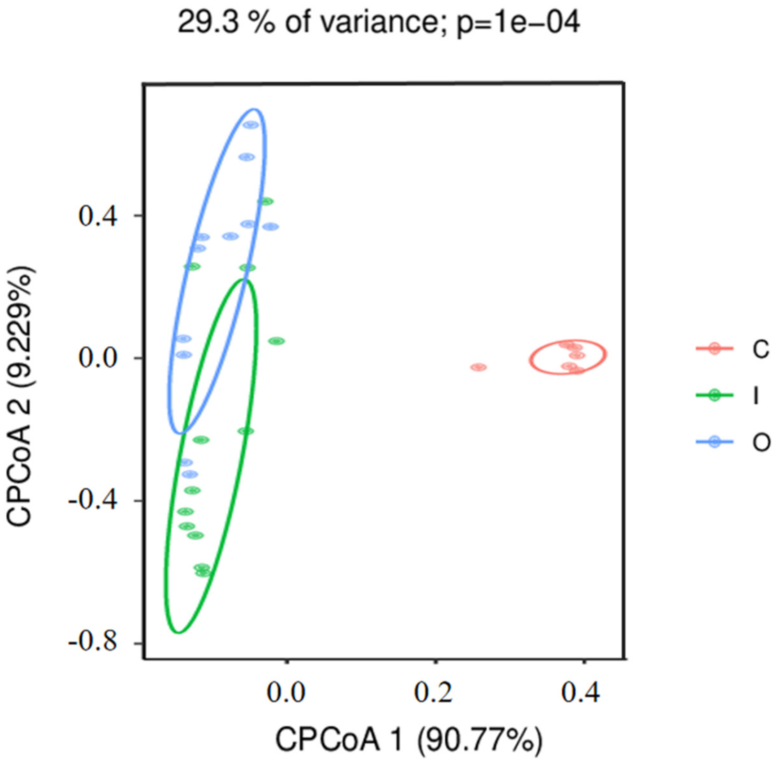

Figure S4 Cluster analysis results of fungal community in samples from compost piles (C), inside (I) and outside (O) composting workshop.

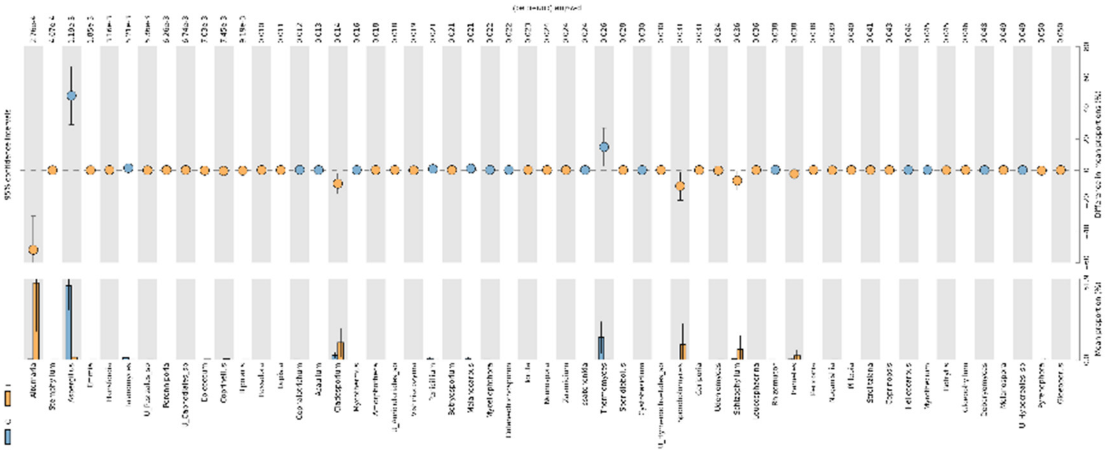

Figure S5. Identification of different fungal genera between compost piles (C) and inside composting workshop (I) using Stamp analysis. The bar chart depicts the average proportion of differential pathogen/allergen containing genera predicted using Picrust2. The difference in proportions between any two groups is presented with 95% confidence intervals. Only P values < 0.05 (Welch's t-test, FDR adjusted) are depicted.

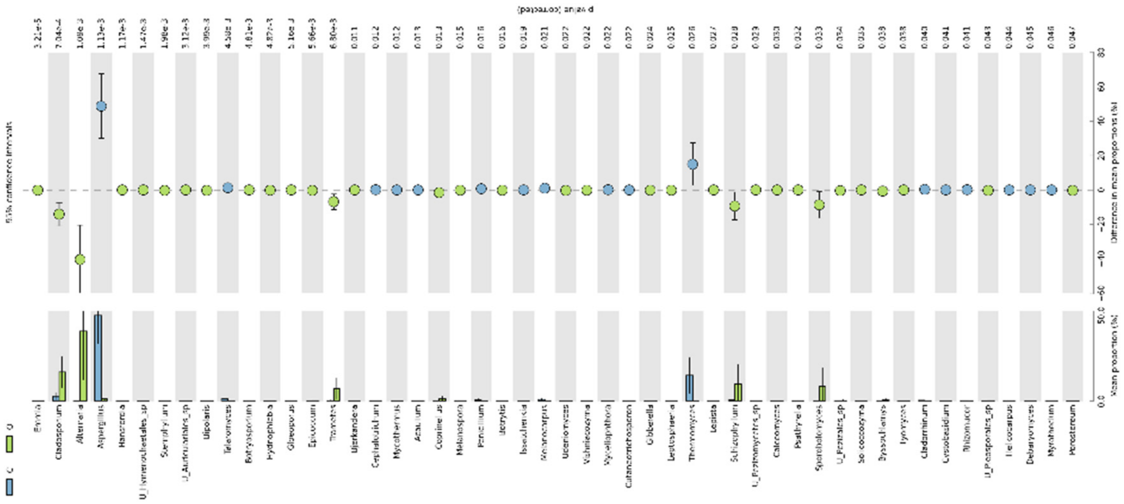

Figure S6. Identification of different fungal genera between compost piles (C) and outside composting workshop (O) using Stamp analysis. The bar chart depicts the average proportion of differential pathogen/allergen containing genera predicted using Picrust2. The difference in proportions between any two groups is presented with 95% confidence intervals. Only P values < 0.05 (Welch's t-test, FDR adjusted)

are depicted.

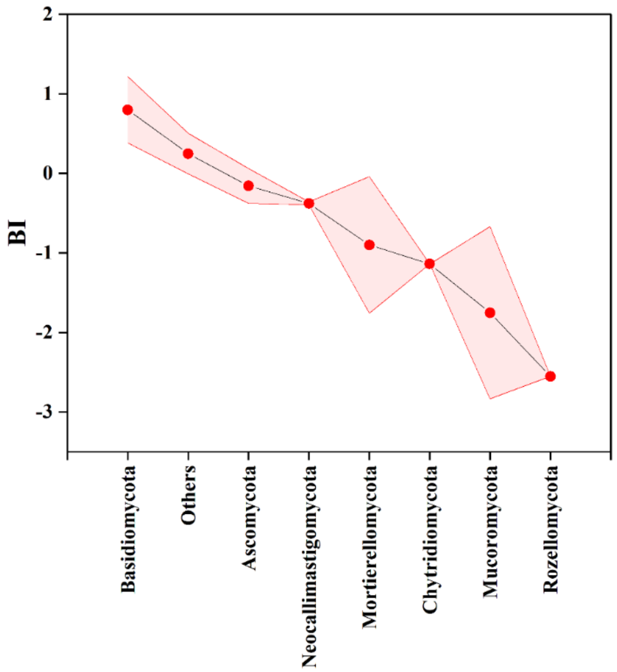

Figure S7. Bioaerosolization Index (BI) of fungal phylum. (BI value was transformed by log)

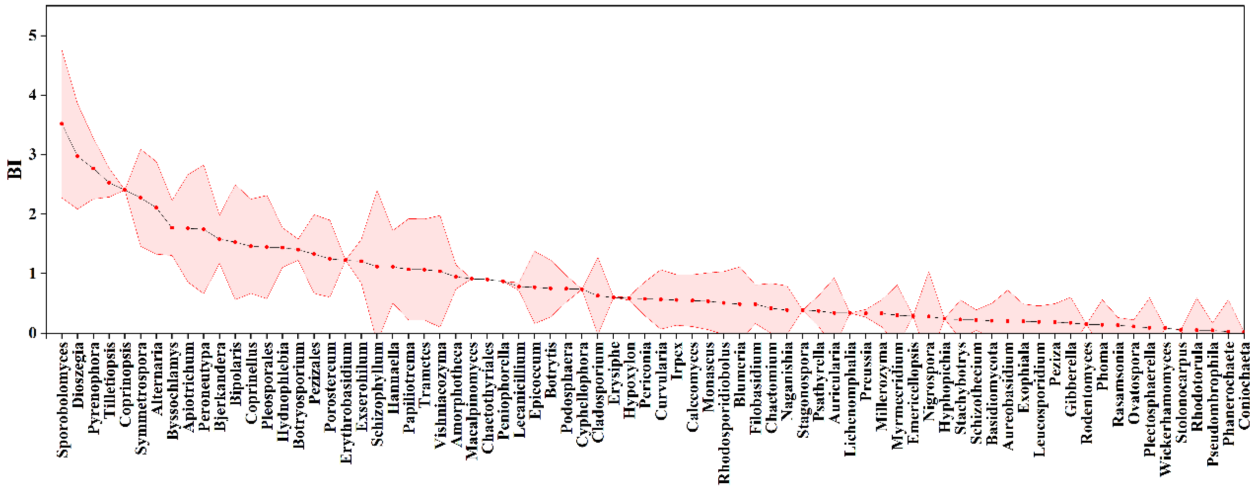

Figure S8. Bioaerosolization Index (BI) of 77 fungal genera with a BI value greater than 0. (BI value was transformed by log)
